# Supplementary material for: Lanthanum Carbonate Opacities—A Systematic Review
Source: Diagnostics (Basel). 2022 Feb 11;12(2):464. doi: 10.3390/diagnostics12020464 (PMC8871341; doi:10.3390/diagnostics12020464)
Supplement: Supplementary file 1 [file diagnostics-12-00464-s001.zip › diagnostics-1586094-supplementary.pdf]

diagnostics

MDPI

Search Strategy

Ovid MEDLINE September 28, 2020

1) Chronic kidney falure.MP or exp Kidney Failure, Chronic/
2) Chronic renal insufficienc*.mp. [mp=title, abstract, original title, nameof substance word, subject heading word, floating sub-heading word,keyword heading word, organism supplementary concept word,protocol supplementary concept word, rare disease supplementaryconcept word, unique identifier, synonyms]
3) Chronic kidney insufficienc*.mp. [mp=title, abstract, original title,name of substance word, subject heading word, floating sub-headingword, keyword heading word, organism supplementary conceptword, protocol supplementary concept word, rare diseasesupplementary concept word, unique identifier, synonyms]
4) exp Renal Insufficiency, Chronic/ or Chronic kidney disease*.mp. orexp Kidney/ or exp Kidney Failure, Chronic/ or exp Kidney Diseases/
5) exp Renal Insufficiency, Chronic/ or exp Kidney Failure, Chronic/ orexp Kidney Diseases/
6) exp Renal Insufficiency, Chronic/ or exp Kidney Failure, Chronic/ orexp Kidney Diseases/ or Chronic Renal disease*.mp. or exp Kidney/
7) exp Acute Kidney Injury/ or exp Renal Dialysis/ or exp KidneyFailure, Chronic/ or exp Kidney Transplantation/ or Chronic kidneyfailure*.mp. or exp Kidney/
8) Chronic renal failure.mp. or exp Kidney Failure, Chronic/
9) exp Kidney Transplantation/ or exp Acute Kidney Injury/ or expRenal Insufficiency, Chronic/ or exp Kidney Failure, Chronic/ or End-stage kidney disease*.mp. or exp Renal Dialysis/
10) End stage kidney disease.mp. or exp Kidney Failure, Chronic/
11) exp Renal Dialysis/ or exp Kidney Failure, Chronic/ or exp KidneyTransplantation/ or end-stage renal disease*.mp. or exp KidneyDiseases/
12) end stage renal disease.mp. or exp Kidney Failure, Chronic/
13) ESRD.mp. or exp Kidney Failure, Chronic/
14) exp Renal Insufficiency, Chronic/ or exp Kidney/ or exp Acute KidneyInjury/ or Chronic kidney disorder*.mp. or exp Kidney Diseases/ orexp Kidney Failure, Chronic/
15) exp Kidney Transplantation/ or exp Kidney Diseases/ or exp RenalInsufficiency, Chronic/ or Chronic nephropathy.mp. or exp Kidney/ or exp Kidney Failure, Chronic/
16) (Chronic kidney disease-mineral and bone disorder*).mp. [mp=title,abstract, original title, name of substance word, subject headingword, floating sub-heading word, keyword heading word, organismsupplementary concept word, protocol supplementary concept word,rare disease supplementary concept word, unique identifier,synonyms]
17) CKD-MBD.mp. or exp "Chronic Kidney Disease-Mineral and BoneDisorder"/
18) (CKD-mineral and bone disorder).mp. [mp=title, abstract, originaltitle, name of substance word, subject heading word, floating sub-heading word, keyword heading word, organism supplementaryconcept word, protocol supplementary concept word, rare diseasesupplementary concept word, unique identifier, synonyms]
19) 1 or 2 or 3 or 4 or 5 or 6 or 7 or 8 or 9 or 10 or 11 or 12 or 13 or 14 or15 or 16 or 17 or 18
20) Lanthanum.mp. or exp Lanthanum/
21) exp Lanthanum/ or fosrenol.mp.
22) 20 or 21
23) 19 and 22
24) limit 23 to (danish or english or german or norwegian or swedish)

Ovid MEDLINE & Embase Classic+Embase September 21, 2020

1) Chronic kidney failure.mp. or exp Kidney Failure, Chronic/
2) Chronic renal insufficienc*.mp. [mp=title, abstract, original title, nameof substance word, subject heading word, floating sub-heading word,keyword heading word, organism supplementary concept word,protocol supplementary concept word, rare disease supplementaryconcept word, unique identifier, synonyms]
3) Chronic kidney insufficienc*.mp. [mp=title, abstract, original title,name of substance word, subject heading word, floating sub-headingword, keyword heading word, organism supplementary conceptword, protocol supplementary concept word, rare diseasesupplementary concept word, unique identifier, synonyms]
4) exp Renal Insufficiency, Chronic/ or Chronic kidney disease*.mp. orexp Kidney/ or exp Kidney Failure, Chronic/ or exp Kidney Diseases/
5) exp Renal Insufficiency, Chronic/ or exp Kidney Failure, Chronic/ orexp Kidney Diseases/
6) exp Renal Insufficiency, Chronic/ or exp Kidney Failure, Chronic/ orexp Kidney Diseases/ or Chronic Renal disease*.mp. or exp Kidney/
7) exp Acute Kidney Injury/ or exp Renal Dialysis/ or exp KidneyFailure, Chronic/ or exp Kidney Transplantation/ or Chronic kidneyfailure*.mp. or exp Kidney/
8) Chronic renal failure.mp. or exp Kidney Failure, Chronic/
9) exp Kidney Transplantation/ or exp Acute Kidney Injury/ or expRenal Insufficiency, Chronic/ or exp Kidney Failure, Chronic/ or End-stage kidney disease*.mp. or exp Renal Dialysis/

10) End stage kidney disease.mp. or exp Kidney Failure, Chronic/
11) exp Renal Dialysis/ or exp Kidney Failure, Chronic/ or exp KidneyTransplantation/ or end-stage renal disease*.mp. or exp KidneyDiseases/
12) end stage renal disease.mp. or exp Kidney Failure, Chronic/
13) ESRD.mp. or exp Kidney Failure, Chronic/
14) exp Renal Insufficiency, Chronic/ or exp Kidney/ or exp Acute KidneyInjury/ or Chronic kidney disorder*.mp. or exp Kidney Diseases/ orexp Kidney Failure, Chronic/
15) exp Kidney Transplantation/ or exp Kidney Diseases/ or exp RenalInsufficiency, Chronic/ or Chronic nephropathy.mp. or exp Kidney/ or exp Kidney Failure, Chronic/
16) (Chronic kidney disease-mineral and bone disorder*).mp. [mp=title,abstract, original title, name of substance word, subject headingword, floating sub-heading word, keyword heading word, organismsupplementary concept word, protocol supplementary concept word,rare disease supplementary concept word, unique identifier,synonyms]
17) CKD-MBD.mp. or exp "Chronic Kidney Disease-Mineral and BoneDisorder"/
18) (CKD-mineral and bone disorder).mp. [mp=title, abstract, originaltitle, name of substance word, subject heading word, floating sub-heading word, keyword heading word, organism supplementaryconcept word, protocol supplementary concept word, rare diseasesupplementary concept word, unique identifier, synonyms]
19) 1 or 2 or 3 or 4 or 5 or 6 or 7 or 8 or 9 or 10 or 11 or 12 or 13 or 14 or15 or 16 or 17 or 18
20) Lanthanum.mp. or exp Lanthanum/
21) exp Lanthanum/ or fosrenol.mp.
22) 20 or 21
23) 19 and 22
24) limit 23 to (danish or english or german or norwegian or swedish)
Web of Science Core Collection 23.9.2020***
1) TS=lanthanum
2) TS=fosrenol
3) #2 OR #1
4) TS=chronic renal insufficienc*
5) TS=Chronic kidney insufficienc*
6) TS=Chronic kidney disease*
7) TS=CKD
8) TS=Chronic Renal Disese*
9) TS=Chronic kidney failure*
10) TS=Chronic renal failure*
11) TS=End-stage kidney disease*
12) TS= End stage kidney disease*
13) TS=End stage renal disease*
14) TS=End-stage renal disease*
15) TS=ESRD
16) TS=Chronic kidney disorder*
17) TS=Chronic nephropathy
18) TS= Chronic kidney Disease mineral bone disorder
19) TS=CKD-MBD
20) TS=CKD mineral bone disorder
21) TS=renal dialysis
22) TS=renal dialyses
23) TS=hemodialysis
24) TS=haemodialysis
25) TS=Hemodialyse
26) TS=hemorenodialysis
27) TS=Extracorporeal dialysis
28) TS= Extracorporeal dialyses
29) TS=hemodiafiltration
30) TS=haemodiafiltration
31) TS=Acetate-free biofiltrations
32) TS=Acetate-free biofiltration
33) TS=blood dialysis
34) TS=dialysis, blood
35) TS=extracorporeal blood cleansing
36) TS=extracorporal dialysis
37) TS=CVVHDF

38) TS= home dialysis
39) TS=haemodialysis, home
40) TS=hemodialysis, home
41) #40 OR #39 OR #38 OR #37 OR #36 OR #35 OR #34 OR #33 OR #32 OR #31 OR #30 OR #29 OR #28 OR #27 OR #26 OR #25 OR #24 OR #23 OR #22 OR #21 OR #20 OR #19 OR #18 OR #17 OR #16 OR #15 OR #14 OR #13 OR #12 OR #11 OR #10 OR #9 OR #8 OR #7 OR #6 OR #5 OR #4
42) #41 AND #3
43) #41 AND #3
Refined by: [excluding] RESEARCH AREAS: (BUSINESS ECONOMICS OR ENGINEERING OR VETERINARY SCIENCES OR CELL BIOLOGY OR ENVIRONMENTAL SCIENCESECOLOGY)
*** Indexes=SCI-EXPANDED, SSCI, A&HCI, CPCI-S, CPCI-SSH, ESCI Timespan=All years
